# Prevalence and risk indicators of non-carious cervical lesions in male footballers

**DOI:** 10.1186/s12903-020-01200-9

**Published:** 2020-07-29

**Authors:** Tamea Lacerda Monteiro Medeiros, Sheila Cristina Almeida Neves Mutran, Daybelis González Espinosa, Kelson do Carmo Freitas Faial, Helder Henrique Costa Pinheiro, Roberta Souza D’Almeida Couto

**Affiliations:** 1 School of Dentistry, Federal University of Pará, Augusto Corrêa Street 937 – odd side, Guamá, Belém, PA 66075110 Brazil; 2 Evandro Chagas Institute, BR316 - km7, Marituba, Pará Brazil

**Keywords:** Non-carious cervical lesions, Athlete, Saliva, Dentin

## Abstract

**Background:**

Non-carious cervical lesions (NCCLs) have shown a significant incidence and prevalence and have been increasingly associated with people’s lifestyles and youths. This cross-sectional study aimed to determine the prevalence of NCCLs in footballers and to address potential risk indicators.

**Methods:**

Fourty-three male semi-professional footballers with an average of 27 years old completed a questionnaire and were subjected to intraoral examination in terms of cervical tooth wear, morphological characteristics of NCCLs, tooth sensitivity, occlusal/incisal wear, and malocclusion classification. Also, laboratory assays were performed to determine salivary parameters: flow rate, pH, buffer capacity, level of Ca (calcium), Na (sodium), and K (potassium) ions, and level of cortisol. The data obtained from the questionnaire and intraoral examinations were subjected to Chi-square and Poisson regression models while the data obtained from the laboratory assays were analyzed by using analysis of variance (*p* <  0.05).

**Results:**

The prevalence of NCCLs was 39.5%. The participants presented predominantly initial lesions with signs of mechanical stress. The daily training time was found as a significant risk indicator (*p* = 0.028). The multivariate analysis showed a significant difference in the variables daily training time (*p* = 0.023), lemon water intake while fasting (*p* = 0.002), toothpaste type (*p* = 0.004), tooth sensitivity (*p* = 0.006); previous orthodontic treatment (*p* = 0.003), and occlusion type (*p* = 0.008). All participants presented normal salivary parameters and levels of cortisol.

**Conclusion:**

The prevalence of NCCLs among footballers was remarkable. The premolars were the most affected teeth and presented symptoms/signs of initial lesions. The daily training time was a dominant risk indicator of NCCLs development. Footballers presented adequate salivary parameters and cortisol levels.

## Background

Non-carious cervical lesions (NCCLs) are defined as the loss of dental hard tissue at the cementoenamel junction (CEJ) region without the action of microorganisms or inflammatory processes. These lesions vary from shallow saucer-shaped grooves to deep wedge-shaped defects and can occur sub- or supragingival at facial, lingual, and/or interproximal surfaces. Although this oral disease has a high incidence and prevalence regardless of age, gender, social, economic, and cultural conditions, it has been increasingly associated with people’s lifestyles and youths [1–5]. NCCLs have a multifactorial etiology, including mechanical stress (tension), erosion (chemical degradation), and friction. Risk factors such as teeth clenching, premature or eccentric contacts, overbrushing, and acidic beverages intake modulate the evolution of NCCLs according to their intensity, duration, and frequency.

The combination of factors seems relevant in the formation of NCCLs and mechanical stress is observed in most cases [6]. Individuals with lifestyles that enhance this peculiar interaction between tension, erosion, and friction are more vulnerable to NCCLs [7]. The intense physical exercises and high workloads that athletes are constantly submitted to increase the risk of body and dental trauma [8]. The fluid and electrolytes lost through sweat may cause dehydration and reduce the salivary flow rate. A dry mouth without the protection of saliva combined with acidic sports beverages intake significantly contributes to dental erosion [8–10]. Therefore, this study aimed to determine the prevalence of NCCLs in footballers and to address potential risk indicators.

## Methods

### Participants and ethical aspects

After receiving approval by the Ethics Committee of the Federal University of Pará (CAEE 86808318.2.0000.0018), male semi-professional footballers with at least 20 sound teeth (except for third molars) and not under orthodontic treatment were voluntarily enrolled in this cross-sectional study and signed a written informed consent form. Forty-five players of a football team represented the convenience sampling; therefore, considering an anticipated frequency of 50% at 95% confidence interval, the minimum sample size of 41 was determined with an open-source epidemiologic calculator (OpenEpi 3.01, SSPropor, Emory University, USA). Forty-three participants aged between 18 and 49 years (average of 27 years old) were evaluated from August to November 2018 at the School of Dentistry.

### Questionnaire

The participants completed a questionnaire with regard to their name, gender, age, e-mail address, contact number, education level, occupation, daily training time, and years of a sports activity. The second part of the questionnaire addressed potential indicators of NCCLs development such as oral hygiene regime (toothbrush type, toothpaste, and brushing immediately after meals), medical conditions (dry mouth, gastroesophageal reflux, use of medication), diet (intake of acidic beverages), parafunctional habits (teeth clenching/grinding, nail or object biting), temporomandibular joint (TMJ) pain, tooth sensitivity, and previous orthodontic treatment (supplementary file).

### Intraoral examination

One calibrated evaluator with intraevaluator reliability of 0.84 at the Kappa test performed the intraoral examinations at the School of Dentistry by using mirrors, periodontal, and exploratory probes. The cervical third of each tooth was measured with a periodontal probe to determine the tooth wear index (TWI) of Smith & Knight modified by Soares & Grippo [11]: 0 = absence of NCCL; 1 = shallow NCCL with < 1 mm depth; 2 = moderate NCCL with 1–2 mm depth; 3 = deep NCCL with > 2 mm depth; and R = restoration, darkened surface, fracture, caries or calculus.

The morphological characteristics of NCCLs were classified by a) shape (rounded, wedged, or mixed), considering the angle formed at the pulp wall of the lesion; b) depth (shallow, moderate, and deep), in accordance with the cervical TWI criteria; c) anatomic localization (coronal or apical to the CEJ); d) clinical localization (supra- or subgingival), considering the relation between the apical margin of the lesion and the gingival margin and also the gingival margins of adjacent teeth as parameters; e) dentine aspect (with or without sclerotic dentin); and f) texture (smooth or rough).

The sensitivity level of each tooth was evaluated by using tactile and cold air stimuli. Tactile sensitivity was assessed using a cross-shaped exploratory probe to scratch the tooth surface from apical to occlusal/incisal and from mesial to distal directions. The evaporative stimuli were assessed through a blast of cold air from a triple syringe (40 psi of pressure) applied approximately 2 mm from and perpendicular to the tooth surface. The participants were asked to qualify their sensitivity by using a modified visual analog scale (VAS): 0 = no pain; 10 = most severe pain.

In addition, the teeth occlusal surfaces were photographed and the dental arches were digitalized at maximum intercuspation with aid of an intraoral scanner (TRIOS® Pod, 3Shape, Copenhagen, Denmark). The images were exported to an orthodontics software (OrthoAnalyzer™ 3D, 3Shape Medical, Copenhagen, Denmark) and the occlusal/incisal wear of each tooth was classified in accordance with Mockers and modified by Vieira et al. [12]: 0 = no wear; stage 1 = enamel wear; stage 2 = dentin wear (the occlusal surface with more enamel than dentin; stage 3 = dentin wear (the occlusal surface with more dentin than enamel); or stage 4 = advanced wear stage (either close to or with exposure of the pulp chamber). The bite patterns were classified as Angle Class I, II, and III malocclusions; in addition, alterations such as open-, under-, over-, and crossbite were evaluated [13].

### Laboratory assays

Two saliva samples of each participant were collected in the morning. The first sample was collected with the aid of a specific swab (Salivette tube® Cortisol Sarstedt AG & Co., Nümbrecht, Germany). The level of salivary cortisol was determined through an electrochemiluminescence immunoassay (Cobas e411, Roche Diagnostics International, Basel, Switzerland) at a private laboratory (Paulo C. Azevedo, Belém, Brazil). Samples with concentrations below 20.3 nmol/l were considered normal.

Prior to the second sample collection, the participants chewed a piece of rubber tourniquet for 1 min and discarded the saliva; thus, the participants continued chewing and spit the saliva into a Falcon sterile tube every 1-min interval during 5 min. The second samples were used to determine the salivary parameters at the Laboratory of Biochemistry of the School of Pharmacy of the Federal University of Pará.

Salivary flow rates higher or equal to 0.7 ml/min were considered regular while values lower than 0.7 ml/min determined hyposalivation in accordance with the criteria established by Thylstrup & Fejerskov [14]. The pH of saliva was measured with a pH meter (Thermo Scientific, South Logan, Utah, USA) calibrated with standardized solutions of pH 4, 7, and 10.

The saliva buffering capacity was assessed by actively mixing 1 ml of saliva to 3 ml of 0.005% hydrochloric acid. After 2 min, the recipient was uncovered for 10 min for the evaporation of carbon gas, and the salivary pH was measured with the pH meter. In accordance with Krasse [15], values above or equal to and below 5.5 were classified as regular and low buffering capacity, respectively.

Samples containing 0.5 ml of saliva and 0.5 ml of 10% nitric were prepared, sieved with filter paper, and diluted in 1:10 deionized water. At the Evandro Chagas National Infectology Institute (Belém, Brazil), Ca, Na, and K ions were quantified by using an inductively-coupled plasma optical emission spectrometer (ICP-OES) (Varian Vista-MPX CCD Simultaneous, Varian Analytical Instruments, Mulgrave, Australia), under axial configuration and equipped with an automatic sampling system (SPS-5 Varian Spectrophotometer AutoSampler, Varian Analytical Instruments) and specific software (ICP Expert Vista, Varian Analytical Instruments).

### Statistical analysis

The data obtained from the questionnaire and intraoral examinations were subjected to descriptive analysis, bivariate analysis through the Chi-square test, bivariate and multivariate Poisson regression models (SPSS 2.0, IBM Corp., Chicago, USA). The data obtained from the laboratory assays were analyzed by using analysis of variance (BioEstat 5.0, Mamirauá Institute, Belém, Brazil). A significance level of 95% (*p* <  0.05) was used for all analyses.

## Results

Most of the participants had a high level of education (58.1%), more than two years of a sports activity (62.8%), and more than 1 h of daily training (55.8%). Only 11.6% of the participants reported football as their exclusive occupation. Approximately 79% of the participants reported to use conventional toothpaste, 27.9% used to brush their teeth immediately after meals, 30.2% reported to have dry mouth, 11.6% reported gastroesophageal reflux, 23.2% used medication, 9.3% reported to drink lemon water while fasting, 74.4% reported parafunctional habits, 27.9% reported TMJ pain, 79% presented tooth sensitivity and 30.2% reported previous orthodontic treatment. The majority of the participants (81.4%) presented Angle Class I malocclusion and 41.9% presented an altered bite. The NCCLs were diagnosed in 39.5% of the participants. The footballers that reported up to 1 h of daily training had a significantly higher prevalence of NCCLs than those that trained for more than 1 h per day (*p* = 0.028) (Table 1).

**Table 1:** Descriptive and bivariate analysis of the questionnaire variables

Variables	Total (%)	NCCL+ (%)	NCCL- (%)	*p* - value
Education level				0.638
High School	11 (25.6)	5 (45.5)	6 (54.5)	
University	25 (58.1)	8 (32)	17 (68)	
Postgraduate	7 (16.3)	4 (57.1)	3 (42.9)	
Years of a sports activity				0.834
Up to 2 years	16 (37.2)	6 (37.5)	10 (62.5)	
More than 2 years	27 (62.8)	11 (40.7)	16 (59.3)	
Daily training time				0.028*
Up to 1 h	19 (44.2)	11 (57.9)	8 (42.1)	
More than 1 h	24 (55.8)	6 (25)	18 (75)	
Occupation				0.293
Student	7 (16.3)	3 (42.9)	4 (57.1)	
Worker	18 (41.9)	8 (44.4)	10 (55.6)	
Student and worker	13 (30.2)	6 (46.2)	7 (53.8)	
Without occupation	5 (11.6)	0 (0)	5 (100)	
Toothpaste				0.483
Conventional	34 (79)	12 (35.3)	22 (64.7)	
Desensitizing	3 (7)	2 (66.7)	1 (33.3)	
Whitening	6 (14)	3 (50)	3 (50)	
Brushing immediately after meals				0.383
No	31 (72.1)	11 (35.5)	20 (64.5)	
Yes	12 (27.9)	6 (50)	6 (50)	
Dry mouth				0.146
No	30 (69.8)	14 (46.7)	16 (53.3)	
Yes	13 (30.2)	3 (23.1)	10 (76.9)	
Gastroesophageal reflux				0.319
No	38 (88.4)	14 (36.8)	24 (63.2)	
Yes	5 (11.6)	3 (60)	2 (40)	
Medication				0.149
No	33 (76.8)	15 (45.5)	18 (54.5)	
Yes	10 (23.2)	2 (20)	8 (80)	
Lemon water intake while fasting				0.128
No	39 (90.7)	14 (35.9)	25 (64.1)	
Yes	4 (9.3)	3 (75)	1 (25)	
Parafunctional habits				0.642
No	11 (25.6)	5 (45.5)	6 (54.5)	
Yes	32 (74.4)	12 (37.5)	20 (62.5)	
TMJ pain				0.605
No	31 (72.1)	13 (41.9)	18 (58.1)	
Yes	12 (27.9)	4 (33.3)	8 (66.7)	
Tooth sensitivity				0.735
No	9 (21)	4 (44.4)	5 (55.6)	
Yes	34 (79)	13 (38.2)	21 (61.8)	
Previous orthodontic treatment				0.206
No	30 (69.8)	10 (33.3)	20 (66.7)	
Yes	13 (30.2)	7 (53.8)	6 (46.2)	
Malocclusion				0.763
Angle Class I	35 (81.4)	14 (40)	21 (60)	
Angle Class II	4 (9.3)	1 (25)	3 (75)	
Angle Class III	4 (9.3)	2 (50)	2 (50)	
Bite alteration				0.576
No	25 (58.1)	9 (36)	16 (64)	
Yes	18 (41.9)	8 (44.4)	10 (55.6)	
Total (%)	43 (100)	17 (39.5)	26 (60.5)	

NCCL+: subjects with non-carious cervical lesions. NCCL-: subjects without non-carious cervical lesions. (*) Significant difference (Chi-square test, *p* < 0.05)

The bivariate analysis revealed that the footballers that trained up to 1 h per day (PR 2.32 [1.05–5.11]; *p* = 0.038) and those who consumed lemon water while fasting (PR 2.5 [1.67–3.75]; *p* < 0.001) showed a significant association with the development of NCCLs. The multivariate analysis with adjusted values revealed a significant association between NCCLs and up to 1 h of daily training (PR 3.35 [1.18–9.49]; *p* = 0.023), lemon water intake while fasting (PR 8.10 [2.19–29.90]; *p* = 0.002), use of desensitizing toothpaste (PR 5.30 [1.63–16.96]; *p* = 0.004), presence of tooth sensitivity (PR 2.53 [1.31–4.88]; *p* = 0.006), and previous orthodontic treatment (PR 3.11 [1.48–6.50]; *p* = 0.003) (Table 2).

**Table 2:** Bivariate and multivariate regression analysis of the questionnaire variables

Variables	PR (95% CI)	*p* - value	Adjusted PR (95% CI)	*p* - value
Years of a sports activity				
Up to 2 years	1.09 (0.5–2.37)	0.835	2.74 (0.97–7.72)	0.056
More than 2 years	1		1	
Daily training time				
Up to 1 h	2.32 (1.05–5.11)	0.038*	3.35 (1.18–9.49)	0.023*
More than 1 h	1		1	
Toothpaste				
Whitening	1.29 (0.49–3)	0.684	1.64 (0.56–4.79)	0.369
Desensitizing	1.61 (0.65–4)	0.304	5.26 (1.63–16.96)	0.004*
Conventional	1		1	
Medication				
Yes	0.5 (0.14–1.75)	0.278	0.5 (0.19–1.27)	0.144
No	1		1	
Lemon water intake while fasting				
Yes	2.5 (1.67–3.75)	< 0.001*	8.1 (2.19–29.9)	0.002*
No	1		1	
Tooth sensitivity				
Yes	0.73 (0.34–1.57)	0.427	2.53 (1.31–4.88)	0.006*
No	1		1	
Previous orthodontic treatment				
Yes	1.58 (0.77–3)	0.231	3.11 (1.48–6.5)	0.003*
No	1		1	
Malocclusion				
Angle Class III	1.48 (0.61–3.59)	0.391	0.68 (0.26–1.73)	0.415
Angle Class II	0.55 (0.10–3.16)	0.506	0.13 (0.03–0.59)	0.008*
Angle Class I	1		1	
Bite alteration				
Yes	1.1 (0.54–2.22)	0.795	1.53 (0.77–3.07)	0.226
No	1		1	

*PR* prevalence ratio, *CI* confidence interval. (*) Significant difference (Poisson Regression model, *p* < 0.05)

Occlusal/incisal wear was more frequently observed in anterior teeth (39.7%) and canines (29.1%) followed by premolars (16.3%) and molars (10.9%). The same trend was observed for tooth sensitivity evaluations: anterior teeth (21–24.5%), canines (24.4–23.3-%), premolars (18.1–21.1%), and molars (16.8–15.6%) (Table 3).

**Table 3:** Frequencies and percentages of the intraoral examination variables

	Teeth	Occlusal wear	VAS tactile (+)	VAS air (+)	NCCLs (+)	TWI 1	TWI 2	TWI 3	Class V restoration
Incisors	343 (29.4%)	136 (39.7%)	72 (21%)	84 (24.5%)	4 (1.2%)	4 (1.2%)	0 (0%)	0 (0%)	4 (1.2%)
Canines	172 (14.7%)	50 (29.1%)	42 (24.4%)	40 (23.3%)	12 (7%)	12 (7%)	0 (0%)	0 (0%)	1 (0.3%)
Premolars	331 (28.4%)	54 (16.3%)	60 (18.1%)	70 (21.1%)	56 (16.9%)	53 (16%)	3 (0.9%)	0 (0%)	0 (0%)
Molars	321 (27.5%)	35 (10.9%)	54 (16.8%)	50 (15.6%)	22 (6.8%)	19 (5.9%)	3 (0.9%)	0 (0%)	8 (2%)
Upper	585 (50.1%)	119 (20.3%)	103 (17.6%)	111 (19%)	51 (8.8%)	46 (7.9%)	5 (0.9%)	0 (0%)	7 (1.2%)
Lower	582 (49.9%)	156 (26.8%)	125 (21.5%)	133 (22.9%)	43 (7.4%)	42 (7.2%)	1 (0.2%)	0 (0%)	6 (1%)
Right	584 (50%)	140 (24.0%)	116 (19.9%)	107 (18.3%)	58 (9.9%)	54 (9.2%)	4 (0.7%)	0 (0%)	9 (1.5%)
Left	583 (50%)	135 (23.1%)	112 (19.2%)	137 (23.5%)	36 (6.1%)	34 (5.8%)	2 (0.3%)	0 (0%)	4 (0.7%)
Total	1167 (100%)	275 (23.6%)	228 (19.5%)	244 (20.9%)	94 (8%)	88 (7.5%)	6 (0.5%)	0 (0%)	13 (1.1%)

NCCLs were diagnosed in 8% of the teeth examined (94 of 1167). These lesions were more frequently found in premolars (16.9%), in the upper arch (8.8%), and on the right side (9.9%). The NCCLs were predominantly shallow (< 1 mm depth) and diagnosed in premolars (15.8%). The lesions of moderate depth (1–2 mm) were only observed in premolars (0.9%) and molars (0.9%). Class V restorations were more frequently observed in molars (2%) (Table 3).

Among the teeth with shallow NCCLs (< 1 mm), 83% did not present occlusal/incisal wear and 17% presented wear only in enamel. Occlusal/incisal wear was not observed in those teeth with moderate NCCL (1–2 mm depth) (Table 4). Therefore, the relationship between occlusal/incisal tooth wear and NCCLs depth was not significant (*p* = 0.077).

**Table 4:** Relationship between occlusal/incisal tooth wear and NCCL depth

	NCCL depth				*p* - value
	0	< 1 mm	1–2 mm	Total	
Occlusal/incisal tooth wear					
0	802 (75.6%)	73 (83%)	6 (100%)	881 (76.3%)	0.077
1	213 (20.1%)	15 (17%)	0 (0%)	228 (19.7%)	
2	41 (3.9%)	0 (0%)	0 (0%)	41 (3.6%)	
3	4 (0.4%)	0 (0%)	0 (0%)	4 (0.3%)	
Total	1060 (100%)	88 (100%)	6 (100%)	1154 (100%)	

(*) Significant difference (Chi-square test, *p* ≤ 0.05)

**Fig. 1:**
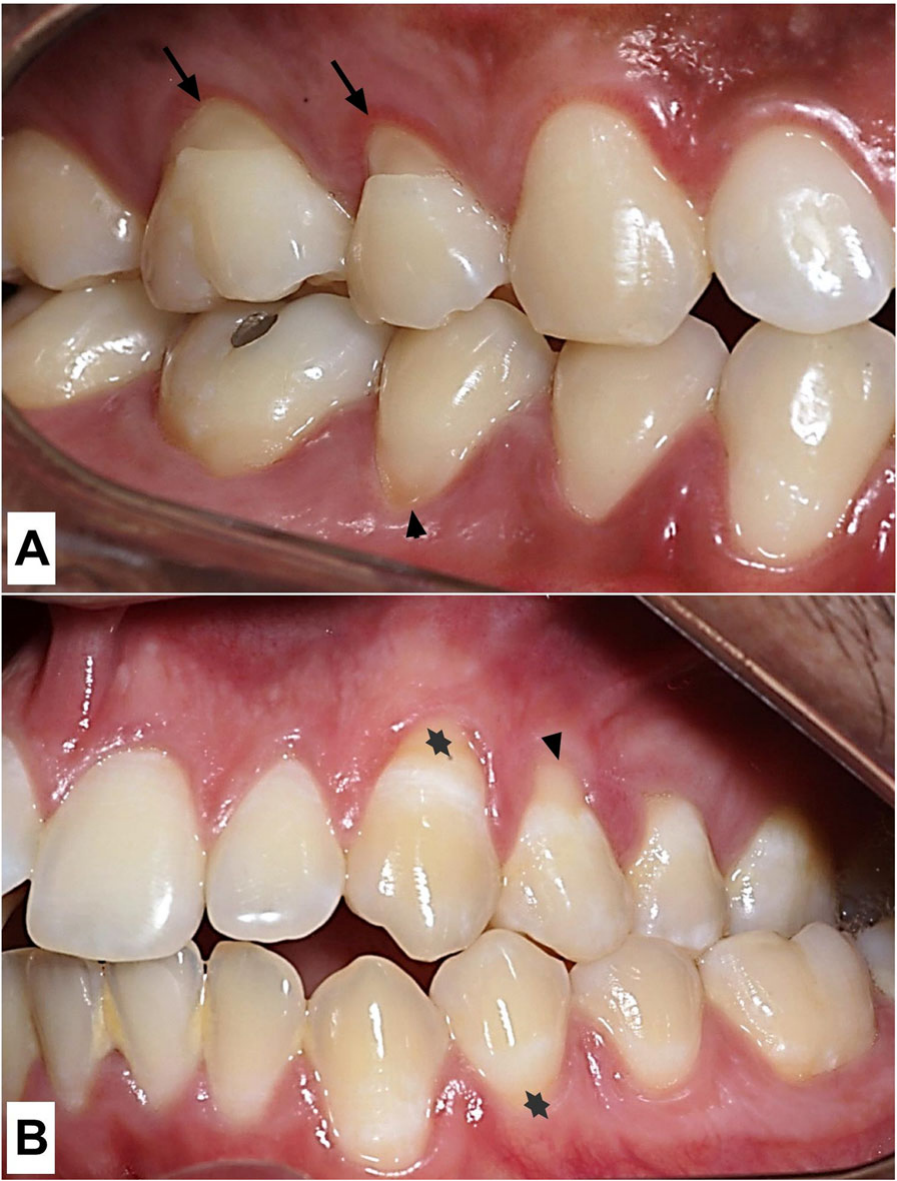
Clinical appearance of NCCLs. **a** The upper black arrows indicates wedge-shaped moderate NCCLs (1–2 mm depth) while the lower black arrowhead shows a rounded shallow NCCL (< 1 mm depth). All lesions are located coronally to the CEJ, present supragingival clinical location, absence of sclerotic dentin and smooth texture. **b** White spots at the cervical teeth outline. The asterisks highlights the white spots (suggestive of cracks) that do not extend to the root in case of gingival recession (black arrowhead)

The most frequent morphological characteristics of the NCCLs were: rounded shape (65%), shallow depth (94%), coronal to the CEJ (52%), supragingival (52%), without sclerotic dentin (98%), and smooth texture (99%). Figure 1 shows the morphological aspects of some NCCLs.

The laboratory assays of participants with or without NCCLs revealed regular salivary flow (higher than 0.7 ml/min), neutral pH, and buffering capacity above 5.5. All participants presented similar amounts of Ca, Na, and K ions, and their level of cortisol remained bellow 20.3 nmol/l. Therefore, none of the salivary analyses indicated significant differences.

## Discussion

This study diagnosed a remarkable prevalence of NCCLs in footballers and revealed the daily training time as an important risk indicator. The NCCLs were diagnosed in 39.5% of the participants, while the data in the literature varies from 5 to 85% due to different types of participants (specific subgroups of the general population) combined with the lack of method standardization [2, 16]. For instance, a study involving Brazilians between 40 to 60 years old reported NCCLs prevalence of 67% while a population from 35 to 74 years old in China presented a prevalence of 81.3% [17, 18]. However, a recent clinical trial with patients between 18 and 40 years old treated for NCCLs and cervical dentin hypersensitivity at a Brazilian university revealed a prevalence of 88.1% and also suggested an increasing distribution of NCCLs among youths [1]. Other studies reported prevalence ratios below 53% [19–23].

The bivariate regression analysis revealed that daily training time is a dominant risk indicator for the development of NCCLs. The fact that footballers that reported only up to 1 h of daily training presented more NCCLs may be related to poor physical conditioning. Therefore, the footballers that trained more than 1 h per day seem to better withstand the intensity and directions of the loads that teeth are subjected to. Similarly, Antunes et al. observed that the training time of Brazilian amateur runners represented a risk factor for dental erosion [22]. Approximately 52% of the runners that expended only up to 1 h during training presented tooth erosion, while the rate decreased to 38 and 9.5% for those runners that trained up to 2 and 3 h, respectively. In addition to good physical conditioning, individuals dedicated to long training times possibly take better healthcare. The intake of lemon water while fasting was significantly associated with NCCLs. Acids of either exogenous (some fruits and juices, carbonated soft drinks, acidic sports beverages, alcoholic drinks, some medications, and occupational factors such as chlorine used in swimming pools) or endogenous origin (gastric juice) contribute to dental erosion and consequently to the development of NCCLs [24–29].

The multivariate regression analysis significantly associated the prevalence of NCCLs with several variables (daily training time, lemon water intake while fasting, use of desensitizing toothpaste, tooth sensitivity, and previous orthodontic treatment), which confirms that the combination of factors is more relevant in the development of these lesions [1, 7].

Our findings reinforce that the indiscriminate use of desensitizing toothpaste without professional follow-up impairs the early diagnosis of NCCLs since the painful symptoms are masked and the individual does not seek appropriate treatment. The presence of tooth sensitivity allows proper NCCL diagnosis and its association with other variables favors the development of the lesions [30].

Although orthodontic treatment induces stress in the cervical region of teeth, the intensity of the force applied by orthodontic appliances should not exceed the intrinsic tensile and compression strength of dental structures [31, 32]. Spini assessed the cervical tension of premolars through finite element analysis and extensiometry and concluded that the accumulation of tension and deformity was higher in teeth with NCCL and previously subjected to orthodontics [33]. In our study, the significant association between orthodontic treatment and NCCLs may be explained by the excessive forces that teeth were subjected during successive years and caused structural losses of enamel and alveolar bone. The increasing prevalence of NCCLs among youths represents novel information to the literature. However, it was observed that most participants did not have their orthodontic treatment completed.

Brandini et al. [19] and Kolak et al. [20] evaluated men and women with a similar age range and diagnosed NCCLs in 13.2 and 3.1% of teeth, respectively. In comparison with the abovementioned studies, our prevalence of NCCLs in the examined teeth remained in the intermediate percentage level of 8% and the lesions were more frequently found in upper premolars. It is a consensus in the literature that premolars present the highest prevalence of NCCLs due to an unfavorable crown-root proportion and the thin buccal bone plate. These features result in a high-stress concentration in the cervical region during eccentric mandibular movements, particularly in the case of group disocclusion [1, 17, 19, 20, 34–37].

The diagnosed NCCLs in this study were predominantly shallow, rounded, supragingival, and without sclerotic dentin, which characterizes them as initial lesions [38]. However, many participants also presented white spots at the cervical teeth outline that does not extend to the root in case of gingival recession. The white color does not change whether the enamel is wet or dry and the concentration varies from diffuse to a well-defined horizontal line. Considering that the footballers are constantly subjected to either tension or mechanical stress, we diagnosed these spots as cracks. This is supported by the concept of striations, which were defined as irregular horizontal enamel bands in the cervical region affected by molecular decomposition (molecular slips or Luder lines) [39]. Lee and Eakles [40] stated that when hydroxyapatite crystals are ruptured, the spaces are filled with water molecules from saliva that impair chemical bonds between crystals and the tooth becomes more vulnerable to mechanical and erosive damages. The water penetration into the dental structure may explain such a whitish aspect and the different shades may be related to the crack depth.

Posterior teeth showed the highest and lowest frequencies of tooth sensitivity and occlusal wear, respectively. Interestingly, the opposite trend was observed for incisors and canines; however, no correlation was found. The different number of participants with or without NCCLs figures as a limitation of this study that reduces the statistical efficiency.

The relationship between the occlusal/incisal wear and NCCL depth was inverse. Among the 94 teeth with NCCLs, 79 did not present occlusal/incisal wear, and 15 presented exclusive enamel wear. Senna et al. [41] conducted a systematic review on the association between NCCLs and occlusion and suggested that occlusal wear is a nature’s way to eliminate occlusal disturbances. The authors also reported that NCCLs develop before or during the establishment of occlusal wear when the teeth are subjected to occlusal stress. Despite the role of tension in the development of NCCLs, an inversive relationship was observed in our study since footballers with NCCLs showed little or no occlusal/incisal wear.

It was observed that the incidence of positive sensitivity in premolars (18.1 to 21.1%) was higher than the incidence of NCCLs (16.9%). We concluded that tooth sensitivity combined with the presence of white spots (cracks induced by stress) in many participants is symptoms/signs of initial NCCL.

The laboratory assays indicated normal salivary parameters in those participants with NCCLs; therefore, these lesions seem related to excessive mechanical stress and not to erosive factors such as acidic beverages or brushing immediately after meals. Fullagar et al. reported that footballers possess limited nutrition and hydration practices [42]. The level of cortisol of all participants was found normal. Although cortisol is regarded as a stress biomarker, the level of anxiety in footballers varies greatly depending on their physical performance [43–45]. After evaluating both trained and untrained men, Rimmele et al. reported that physical activity may provide a protective effect against disorders related to stress since it regulates the level of cortisol [46].

Despite the symptoms/signs of initial NCCLs, the examined footballers need preventive or even curative treatment to control the progression of the lesions. Moreover, periodic follow-ups of their salivary and nutritional conditions are important to dental health. Due regard must be given to the daily training time as an important risk indicator of NCCLs development since the contemporary lifestyle of youths includes intensive physical exercises and frequent intake of acidic sports beverages. Therefore, clinicians must investigate and guide patients to reduce the risk of NCCLs development.

## Conclusion

The prevalence of NCCLs among footballers was remarkable. The premolars were the most affected teeth and presented symptoms/signs of initial lesions. The daily training time was a dominant risk indicator of NCCLs development. These lesions were also significantly associated with several indicators (daily training time, lemon water intake while fasting, use of desensitizing toothpaste, tooth sensitivity, and previous orthodontic treatment). Footballers presented adequate salivary parameters and cortisol levels.

## Supplementary information

**Additional file 1: Supplementary file:** Questionnaire of socioeconomic status, medical history and habits. The questionnaire has been developed for this study.

## Data Availability

The datasets used and/or analysed during the current study are available from the corresponding author on reasonable request.

## Abbreviations

NCCLs: Non-carious cervical lesions
Ca: Calcium
Na: Aodium
K: Potassium
CEJ: Cementoenamel junction
TMJ: Temporomandibular joint
TWI: Tooth wear index
mm: Millimeter
psi: Pound per square inch
VAS: Visual analog scale
nmol/l: Nanomoles per liter
min: Minutes
ml: Milliliter
ICP-OES: Inductively-coupled plasma optical emission spectrometer
PR: Prevalence ratio
CI: Confidence interval
