# Supplementary material for: Prevalence and risk indicators of non-carious cervical lesions in male footballers
Source: BMC Oral Health. 2020 Jul 29;20:215. doi: 10.1186/s12903-020-01200-9 (PMC7392645; doi:10.1186/s12903-020-01200-9)
Supplement: Supplementary file 1 — Additional file 1: Supplementary file: Questionnaire of socioeconomic status, medical history and habits. The questionnaire has been developed for this study. [file 12903_2020_1200_MOESM1_ESM.docx]

| **Questionnaire of socioeconomic status, medical history and habits** |
| --- |
| Name: Gender: Date of birth: Participant number: |
| Phone number: E-mail: Facebook/Instagram: |
| 1) Do you study? ( ) Yes ( ) No |
| 2) What is your education level?  ( ) Preprimary ( ) Elementary School ( ) High School ( ) College ( ) Post-graduation |
| 3) Do you work? ( ) Yes ( ) No |
| 4 ) How do you consider your oral health? ( ) Excellent ( ) Very good ( ) Good ( ) Regular ( ) Bad |
| 5) Do you have tooth sensitivity? ( ) Never ( ) Seldom ( ) Sometimes ( ) Always |
| 6) Have you already been to the dentist? ( ) If yes, skip to question 7 ( ) If no, skip to question 11 |
| 7) Where do you usually seek dental care?  ( ) Private dentist ( ) Health insurance ( ) Public healthcare facility ( ) Public school  ( ) Public university ( ) I do not know / remember ( ) Other: _____________________ |
| 8) When was your last dental appointment?  ( ) I am in treatment ( ) < 6 months ( ) 7 – 12 months  ( ) 13 – 24 months ( ) > 24 months ( ) I do not remember |
| 9) What was the reason for your last dental appoitment?  ( ) Pain ( ) Tooth extraction ( ) Restoration ( ) Tooth sensitivity  ( ) Prophylaxis (cleaning, fluoride, etc) ( ) Other: ____________________ |
| 10) What is the most frequent reason?  ( ) Regular follow-up ( ) Only when I have a problem ( ) I do not know / remember |
| 11) Select the option that best describes the reason made it difficult for you to seek dental care:  ( ) Fear ( ) Cost of treatment and/or transportation ( ) Lack of time ( ) No dentist available  ( ) Lack of confidence in the dentist ( ) Lack of confidence in the treatment benefit  ( ) Dissatisfaction with previous treatments ( ) Other: _______________________________  ( ) I do not know / remember |
| 12) How long have you been playing football?  ( ) < 1 year ( ) 1 – 2 years ( ) 2 – 3 years ( ) 3 – 4 years ( ) > 4 years |
| 13) What is your daily training time?  ( ) 30 min ( ) 45 min ( ) 1 hour ( ) 1 hour and 30 min ( ) 2 hours ( ) > 2 hours |
| 14) Which beverage(s) do you drink more than once a day?  ( ) Water ( ) Energy drink ( ) Soft drink ( ) Wine ( ) Orange juice ( ) Cupuaçu juice ( ) Bier ( ) Coffee |
| 15) Do you drink lemon water while fasting? ( ) Yes ( ) No  If yes, when do you brush your teeth? ( ) Before ( ) After |
| 16) What do you hydrate yourself during training?  ( ) Water ( ) Energy drink ( ) Anything ( ) Other: __________________ |
| 17) Do you feel your mouth dry during training? ( ) No ( ) Only before training ( ) Yes |
| 18) Which type of toothbrush do you use? ( ) Soft ( ) Medium ( ) Hard |
| 19) Which type of toothpaste do you use?  ( ) Conventional ( ) For sensitive teeth ( ) With whitening effect ( ) For gum disease |
| 20) Do you brush your teeth immediately after meals? ( ) Yes ( ) No |
| 21) Do you use mouthguard during training? ( ) No ( ) Yes |
| 22) Have you ever undergone orthodontic treatment? ( ) No ( ) Yes ( ) Yes, but not unfinished |
| 23) Do you use any medication? ( ) Vitamins ( ) Hormones ( ) Other: ____________________ |
| 24) Do you have any of the following habit(s)?  ( ) Teeth clenching at daytime ( ) Bruxism while sleeping ( ) Nail biting ( ) Object biting ( ) None |
| 25) Do you feel muscular pain in the region of the ears and cheeks? ( ) Yes ( ) No |
| 26) Have you been submitted to bariatric surgery? ( ) Yes ( ) No |
| 27) Do you feel nauseous at daytime? ( ) Yes ( ) No |
| 28) Do you have gastroesophageal reflux? ( ) Yes ( ) No |
